# Complete genome sequence of *Planococcus* sp. PAMC21323 isolated from Antarctica and its metabolic potential to detoxify pollutants

**DOI:** 10.1186/s40793-018-0334-y

**Published:** 2018-11-09

**Authors:** Jong-Hyun Jung, Min-Ho Joe, Dong-Ho Kim, Hyun Park, Jong-il Choi, Sangyong Lim

**Affiliations:** 1 Research Division for Biotechnology, Korea Atomic Energy Research Institute, Jeongeup, 56212 Republic of Korea; 2 Department of Radiation Biotechnology and Applied Radioisotope Science, University of Science and Technology, Daejeon, 34113 Republic of Korea; 3 Korea Polar Research Institute, Incheon, 21990 Republic of Korea; 4 Department of Biotechnology and Bioengineering, Chonnam National University, Gwangju, 61186 Republic of Korea

**Keywords:** Planococcus, Antarctica, Psychrophiles, Bioremediation

## Abstract

The *Planococcus* sp. PAMC21323 is a yellow pigment-producing bacterium isolated from King George Island in Antarctica; it has a broad growth temperature range of 5–40 °C. Herein, we describe the complete genome sequence information of the genus *Planococcus* with its annotated sequence, genetic features for bioremediation, and oxidative stress capacity. The *Planococcus* sp. PAMC21323 possesses chromosomal DNA (3,196,500-bp) with plasmid DNA (3364-bp). The complete 3,199,864-bp of the genome consists of 3171 genes including 60 transfer RNAs and 24 ribosomal RNAs. Strain PAMC21323 encodes various genes associated with detoxification of heavy metal ions and aromatic hydrocarbons. Moreover, it is equipped with diverse stress response systems, which can be used to sense the internal and oxidative stresses caused by detoxification. This is the first report highlighting the genetic potential of *Planococcus* sp. PAMC21323 in bioremediation, suggesting application of this psychrotrophic strain in bioremediation in harsh environments.

**Electronic supplementary material:**

The online version of this article (10.1186/s40793-018-0334-y) contains supplementary material, which is available to authorized users.

## Introduction

Increasing environmental pollution caused by industrial and other anthropogenic activities has become a major threat to the survival of living organisms. Microorganism-mediated degradation of pollutants such as hydrocarbons and heavy metal ions into non- or less-hazardous substances is an inexpensive and efficient method for clean-up and restoring contaminated areas, hence the applications of various microorganisms for bioremediation, such as *Pseudomonas*, *Burkholderia* and *Rhodococcus*, have been a focus of numerous studies [1]. During the detoxification of pollutants, cells are exposed to abundant reactive oxygen species (ROS) [2]. Therefore, strong stress resistance of the host organism can help improve bioremediation capacity. The cold-adapted bacteria are generally equipped with diverse stress response systems owing to the fact that the cold environment is a major cause of multiple stresses such as osmotic, alkali, and oxidative stress [3]. Consequently, particular interest has arisen in regard to the bioremediation ability of psychrotrophs and psychrophiles [4, 5]. Polar regions, including Antarctica, are putative reservoirs of genetic resources for bioremediation. It has been reported that diverse bacteria isolated in Antarctica are resistant to multiple metal ions [6] and can degrade hydrocarbons [7]. Moreover, cold-adapted bacteria can be used to remove contaminants in cold terrestrial sites where mesophilic microorganisms do not survive [4].

*Planococcus* spp. are gram-positive (+) bacteria in the family of *Planococcaceae* (*Bacillales*, *Firmicutes*)*.* This genus had previously been categorized as *Micrococci*, but the motile cocci in the genus *Micrococcus* was reclassified as the genus *Planococcus* by Migula in 1894, and its chemosystematic properties were demonstrated by Kocur et al. [8]. To date, 18 type strains have been characterized. Most *Planococcus* spp. are predominantly found in cold marine environments. They account for 5.8% of the total bacterial community in the Arctic permafrost [9] and can survive in high salinity regions such as Arctic spring channels [10].

Within the genus, *Planococcus halocryophilus* is known to be tolerant to high levels of salinity (19% NaCl) and grows under subzero temperature (˗10 °C) [9]. The genome analysis of *P. halocryophilus* Or1 shows that it harbors cold- and osmotic-specific mechanisms and multiple copies of isozymes to maintain the cellular system in harsh conditions [11]. Interestingly, some *Planococcus* spp. exhibit heavy-metal resistance and are capable of degrading linear alkanes or aromatic hydrocarbons [12, 13]. The *Planococcus* sp. S5 grows on salicylate or benzoate and also produces a catechol 2, 3-dioxygenase that shows high reactivity toward 4-chlorocatechol [12]. The haloalkaliphilic bacterium *Planococcus* sp. ZD22 can not only degrade benzene, toluene, xylene, and halogenated benzene, but also use them as sole carbon source [13]. These examples demonstrate that *Planococcus* spp. are credible candidates for utilization in bioremediation resource processes in harsh conditions. However, there have been no reports of the genome features associated with bioremediation pathways, even though 10 genomes of *Planococcus* spp. have been sequenced to date. Many studies have focused on adaption mechanisms of the *Planococcus* spp. under high salt environments or subzero conditions [11].

In this study, we present the complete genome sequence of the psychrotroph *Planococcus* sp. PAMC21323, isolated from King George Island of the South Shetland Islands in Antarctica (62°07′48″ S, 58°28′12″ W), and its genetic properties associated with pollutant degradation and stress resistance.

## Organism information

### Classification and features

**Fig. 1:**
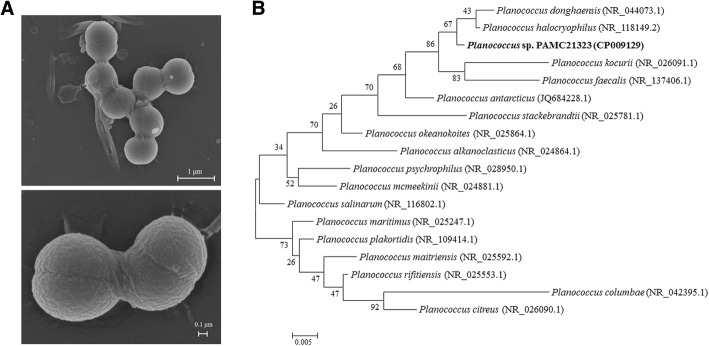
Scanning electron micrograph of *Planococcus* sp. PAMC21323 (**a**) and Phylogenetic analysis of Planococcus sp. PAMC21323 relative to nearest-neighboring *Planococcus* type strains (**b**): The 16 s sequences were obtained from the NCBI database and aligned using ClustalOmega. Phylogenetic tree constructed with the Maximum-Likelihood algorithm of MEGA 6.0. Bootstrap values were determined from 1000 replicates

*Planococcus* sp. PAMC21323 is a gram (+), motile, psychrotrophic bacteria, which can grow over a broad temperature range (5–40 °C). Microscopically, it is a cocci-shaped bacterium measuring 0.5 to 0.7 μm in diameter (Fig. 1a). Colonies are round and yellow in color. The general features of *Planococcus* sp. PAMC21323 are shown in Table 1. Based on multiple alignments of 16S ribosomal RNA (rRNA) sequences of *Planococcus* type strains and *Planococcus* sp. PAMC21323, a phylogenetic tree was constructed using neighbor-joining methods of the MEGA5 program [14] with 1000 bootstrap replicates. *Planococcus* sp. PAMC21323 appeared to represent a phylogenetically coherent group with *P. halocryophilus* and *Planococcus donghaensis* (Fig. 1b)*.* BLASTN analysis revealed that the 16S rRNA sequence of these strains shared 99% similarity.

**Table 1:** Classification and general features of *Planococcus* sp. PAMC21323 according to the MIGS recommendation [42]

MIGS ID	Property	Term	Evidence code^a^
	Classification	Domain *Bacteria*	TAS [43]
		Phylum *Firmicutes*	TAS [43, 44]
		Class *Bacilli*	TAS [43, 45]
		Order *Bacillales*	TAS [43, 46]
		Family *Planococcaceae*	TAS [8, 46]
		Genus *Planococcus*	TAS [8, 46]
		Species PAMC21323	
	Gram stain	Gram positive	TAS [8]
	Cell shape	Coccus	IDA
	Motility	Motile	IDA
	Sporulation	No spore	IDA
	Temperature range	5-40 °C	IDA
	Optimum temperature	25 °C	IDA
	pH range; optimum	4–8; 7.5	IDA
	Carbon source	Glucose, maltose, sucrose, xylose	IDA
MIGS-6	Habitat	Soil (sea shore)	IDA
MIGS-6.3	Salinity	Up to 10%	IDA
MIGS-22	Oxygen requirement	Aerobic	IDA
MIGS-15	Biotic relationship	Not reported	
MIGS-14	Pathogenicity	Non-pathogenic	NAS
MIGS-4	Geographic location	King George Island, Antarctica	IDA
MIGS-5	Sample collection	July 30, 2004	IDA
MIGS-4.1	Latitude	−62.13000	IDA
MIGS-4.2	Longtitude	−58.4700	IDA
MIGS-4.4	Altitude	9	IDA

^a^Evidence codes – *IDA* Inferred from Direct Assay, *TAS* Traceable Author statement (i.e., a direct report exists in the literature), *NAS* Non-traceable Author statement (i.e., not directly observed for the living, isolated sample, but based on a generally accepted property for the species, or anecdotal evidence). These evidence codes are from the Gene Ontology project [47]

## Genome sequencing information

### Genome project history

*Planococcus* spp. are psychrotrophic bacteria that exhibit high resistance toward salt and cold conditions [10, 15]. Some *Planococcus* species were found to show bioremediation activities, but their genetic features related with bioremediation were not investigated [13]. In this study, we isolated the psychrotrophic *Planococcus* sp. PAMC21323 strain from King George Island in the Antarctic and sequenced the genome to investigate its bioremediation potential and stress resistance capacity. The genome project has been deposited in the Genome Online Database [16], and more detailed information is provided in Table 2. The complete genome sequence of the *Planococcus* sp. PAMC21323 is available in the GenBank database.

**Table 2:** Genome sequencing project information

MIGS ID	Property	Term
MIGS-31	Finishing quality	Finished
MIGS-28	Libraries used	454 3 kb paired end library, Illumina 150 bp paired end library
MIGS-29	Sequencing platforms	454-GS-FLX Titanium Illumina Hiseq 2000
MIGS-31.2	Fold coverage	1874-fold coverage
MIGS-30	Assemblers	gsAssembler 2.6
MIGS-32	Gene calling method	Glimmer 3.02
	Locus_Tag	Plano
	Genbank ID	CP009129, CP009130
	Genbank Data of Release	11/19/2014
	GOLD ID	Gp0101987
	BIOPROJECT	PRJNA256273
	Project relevance	Environmental and biotechnology
MIGS-13	Source material identifier	PAMC21323

### Growth conditions and genomic DNA preparation

The *Planococcus* sp. PAMC21323 was cultivated aerobically at 25 °C in a marine broth medium. The genomic DNA was isolated using a Masterpure^Tm^ Gram Positive DNA Purification Kit (Epicenter, Madison WI, USA), according to the standard protocol of the manufacturer.

### Genome sequencing and assembly

The genome of *Planococcus* sp. PAMC21323 was sequenced based on a hybrid strategy using a Roche 454 GS FLX Titanium and an Illumina HiSeq 2000. An 8-kb paired-end library of 454-pyrosequencing, and a 150-bp paired-end library of Illumina, generated 238,440 and 58,949,907 reads, respectively. The CLCbio Genomics Workbench 6.5 software and the Roche gsAssembler 2.6 were used to assemble 1874-fold coverage data of the genome sequence, generating 2 scaffolds with 18 contiguous sequences (contigs). The gaps between the contigs were closed by polymerase chain reaction (PCR) and Sanger sequencing, yielding a genome size of 3,199,864-bp, which consists of one circular chromosome of 3,196,500-bp and one circular plasmid of 3364-bp. The complete genome sequence of *Planococcus* sp. PAMC21323 has been deposited in the GenBank database under accession number CP009129 (Chromosome) and CP009130 (Plasmid).

### Genome annotation

The open reading frames (ORFs) in the complete genome were predicted using a Glimmer 3.02 and a Rapid Annotation using Subsystem Technology (RAST) server [17]. BLASTP analysis based on a non-redundant database and Clusters of Orthologous Groups of proteins (COGs), InterProScan, Pfam, and TIGRFAM databases, was performed to identify the functionality of ORFs [18, 19]. tRNAscan-SE [20] and HMMER [21] were used to identify the transfer RNA (tRNA) and rRNA, respectively. To examine the mobile elements and genomic island (GI) regions, PHAST [22] and IslandViewer (based on the SIGI_HMM, and IslandPath-DIMOB algorithm) [23] were implemented, respectively. Other miscellaneous features were predicted using TMHMM [24] and SignalP [25].

### Genome properties

**Fig. 2:**
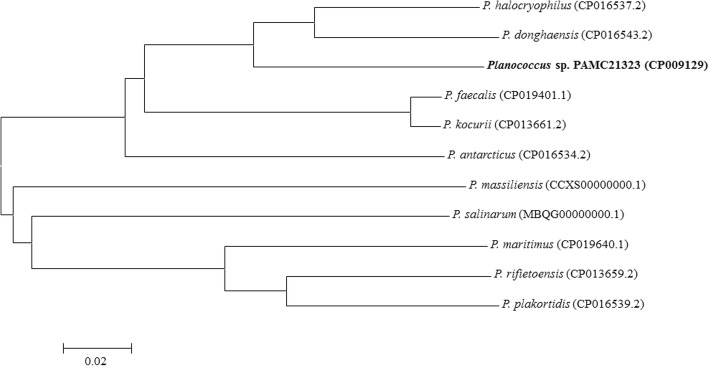
The relationship of the genome sequences of *Planococcus* type strains and PAMC21323 based on the average nucleotide identity values. The bar reflects normalized pairwise genomic distance between genomes

The complete genome of *Planococcus* sp. PAMC21323 consists of chromosomal and extrachromosomal elements with a total length of 3,199,864-bp and GC content of 39.3%. The circular chromosome of 3,196,500-bp (39.3% GC content) was predicted to have 3169 genes, including 60 tRNAs and 24 rRNAs (Table 3). The extrachromosomal element had a length of 3364-bp (33.3% GC content) that encodes two predicted protein-coding genes. Of the total 3171 genes predicted, 3087 were protein-coding genes. The majority (2632 ORF, 85.2%) of all protein-coding genes were assigned with a putative function, whereas the remaining 455 genes were hypothetical proteins. In addition, 2676 ORFs (86.4%) contained at least one or several Pfam domains. The genome summary and COGs categories are listed in Tables 3 and 4. Among the 18 strains identified as a type of the genus *Planococcus*, 10 genome sequences have been registered in the NCBI genome database. The relationship with the other genome sequenced species was calculated based on the average nucleotide identity (ANI) using JSpecies [26]. *Planococcus* sp. PAMC21323 had the highest similarity with *P. halocryophilus* (86.8%) and *P. donghaensis* (86.1%) (Fig. 2). An ANI identity value under 96% shows that PAMC21323 is distinguishable from the other strains.

**Table 3:** Genome statistics of *Planococcus* sp. PAMC21323

Attribute	Value	% of total^a^
Genome size (bp)	3,199,864	100.00
DNA coding region (bp)	2,761,854	86.31
DNA G + C (bp)	1,258,557	39.33
DNA scaffolds	2	–
Total genes	3171	100.00
Protein coding genes	3087	97.35
RNA genes	84	2.65
Pseudo genes	27	0.85
Genes in internal clusters	270	8.51
Gene with function prediction	2632	83.00
Genes assigned to COGs	2294	72.34
Genes assigned Pfam domains	2676	84.39
Genes with signal peptides	128	4.04
Genes with transmembrane helices	840	26.49
CRISPR repeats	1	–

^a^The total is based on either the size of the genome in base pairs or the total number of protein coding genes in the annotated genome

**Table 4:** Number of genes associated with general COG functional categories

Code	Value	% age	Description
J	217	8.26	Translation
A	0	0.00	RNA processing and modification
K	167	6.36	Transcription
L	102	3.88	Replication, recombination and repair
B	1	0.04	Chromatin structure and dynamics
D	42	1.60	Cell cycle control, mitosis and meiosis
Y	0	0.00	Nuclear structure
V	59	2.25	Defense mechanisms
T	128	4.87	Signal transduction mechanisms
M	140	5.33	Cell wall/membrane biogenesis
N	45	1.71	Cell motility
Z	0	0.00	Cytoskeleton
W	8	0.30	Extracellular structures
U	29	1.10	Intracellular trafficking and secretion
O	115	4.38	Posttranslational modification, protein turnover, chaperones
C	131	4.99	Energy production and conversion
G	160	6.09	Carbohydrate transport and metabolism
E	257	9.78	Amino acid transport and metabolism
F	96	3.65	Nucleotide transport and metabolism
H	141	5.37	Coenzyme transport and metabolism
I	148	5.63	Lipid transport and metabolism
P	138	5.25	Inorganic ion transport and metabolism
Q	69	2.63	Secondary metabolites biosynthesis, transport and catabolism
R	255	9.71	General function prediction only
S	167	6.36	Function unknown
X	12	0.46	Mobilome: prophage, transposons
–	877	27.66	Not in COGs

## Insights from the genome sequence

### Diverse mobile genetic elements

**Fig. 3:**
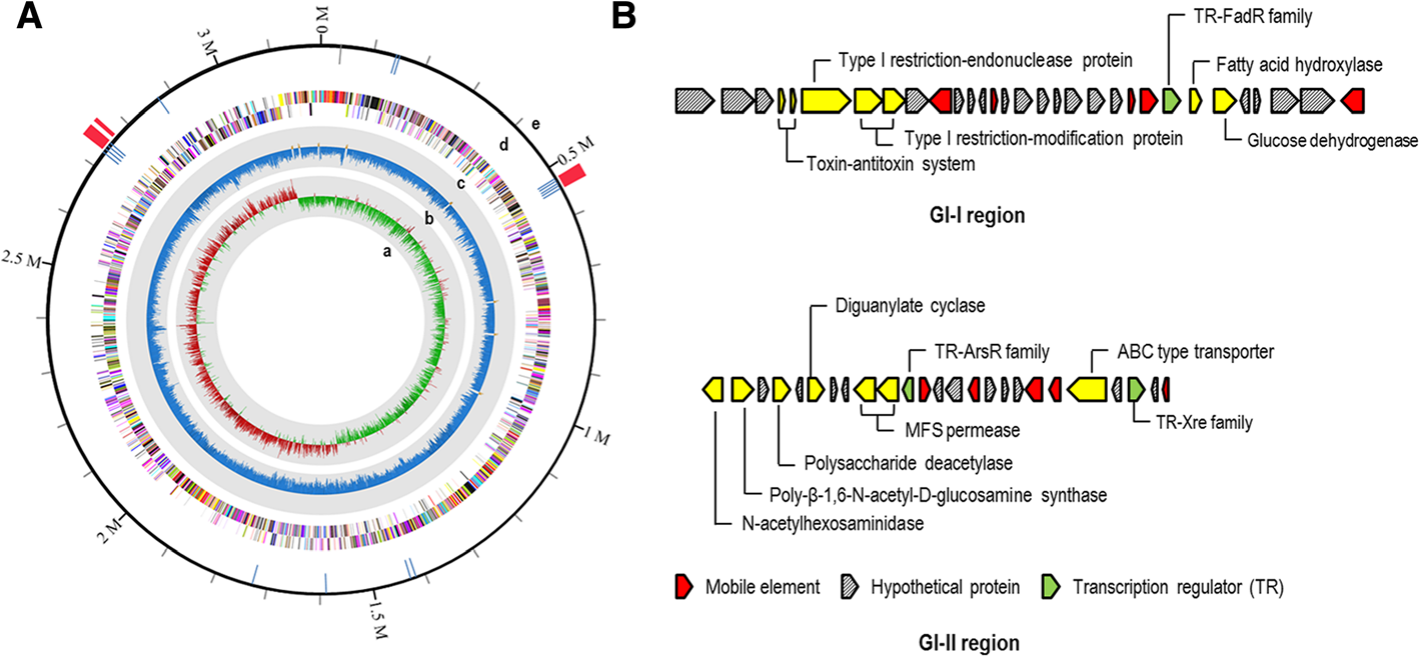
A circular map of the genome of *Planococcus* sp. PAMC21323 (**a**) and gene cluster of genomic islands (**b**). Starting from the inner circle moving outwards, the following tracks are shown: Circle a, positive (red) and negative (green) GC skew; circle b, GC content; circle c, predicted protein coding genes on forward and reverse strand colored to COG categories; circle d, the location of mobile elements (blue); circle e, genomic islands (red)

The mobile elements, such as integrases and transposases, are important genetic components involved in acquisition of new genes, which can expand a genome diversity and adaptation to a new environment [27]. We found that the genome of *Planococcus* sp. PAMC21323 contained 15 putative mobile elements (12 transposases, two integrases, and one Tn552 transposon) known to facilitate horizontal gene transfer (Fig. 3). The number of transposase units found in *Planococcus* sp. PAMC21323 was the same as that in *P. halocryophilus* (12 transposases) but higher than that in *P. donghaensis* (4 transposases) [11]. Interestingly, of the total mobile elements, nine genes were positioned in GI regions, which were identified by IslandViewer 3.0. In the genome of *Planococcus* sp. PAMC21323, three putative GI regions with 24.2 kb, 21.4 kb, and 7.5 kb length, respectively, were observed (Fig. 3). The GI-I region contained five transposase-encoding genes (Plano_0544, 0548, 0556, 0557, and 0566), and four transposase-encoding genes (Plano_2675, 2678, 2683, and 2688) were present in the GI-II region (Fig. 3). Three GI regions account for 1.6% of total chromosomal DNA and include 52 protein coding sequences (Additional file 1: Table S1). Notably, several defense systems were also observed in the GI-I region, one of which was a restriction-modification system (R/M system), a defense system to recognize and remove foreign DNA. Upstream of the R/M system, we found a toxin-antitoxin component (YefM/YoeB family, Plano_0538/0549), which is a stress response module inducing a persistence state that allows cells to cope with different type of stress such as nutrient starvation and temperature stress [28]. The GI-II regions mainly consisted of cell wall modification enzymes, which are known to contribute to cell wall stability and are required to endure osmotic stress [29].

### Genetic features for bioremediation

In the genome of *Planococcus* sp. PAMC21323, various detoxification enzymes for aromatic hydrocarbons, nitroalkanes, and heavy metal ions were identified (Table 5). *Planococcus* sp. PAMC21323 has three extradiol dioxygenases (Plano_0315, 2898, and 2901) that catalyze the cleavage of the aromatic ring structure [30]. Among the enzymes, Plano_2898 and Plano_2901 contain 2, 6-dichloro-*p*-hydroquinone 1, 2-dioxygenase conserved domain (PcpA, pentachlorophenol dioxygenase A), which is probably capable of cleaving aromatic compounds such as γ-hexachlorocyclohexane and 3-nitrophenol. The co-existence of PcpA (2898 and 2901) and PcpB (Plano_2834) shows the possibility that this strain might have the ability to degrade pentachlorophenol, which is an extremely toxic compound in humans, leading to hyperthermia and convulsions [31].

**Table 5:** Identified bioremediation associated genes in PAMC21323

Function	Enzyme	Locus_tag		
Aromatic hydrocarbon degradation	Extradiol dioxygenase	Plano_2898	Plano_2901	Plano_0315
	Pentachlolophenol-4-monooxygenase	Plano_2834		
Nitroalkane degradation	Nitropropane dioxygenase	Plano_2019	Plano_2569	
	Nitroreductase	Plano_0226	Plano_0336	Plano_2301
	Azoreductase	Plano_0380		
Metal ion detoxification	Arsenate reductase	Plano_0840	Plano_1482	Plano_0928
	Mecuric ion reductase	Plano_1475		
Tellurite resistance	TelA	Plano_1607		
	TehB	Plano_1454		

Nitroalkane is a type of organic compound containing a nitro group, which is widely used in industry because it is an intermediate compound in chemical synthesis. It has been known to induce oxidative DNA damage and shown to be carcinogenic [32]. Nitropropane dioxygenase is a member of the nitroalkane oxidizing enzyme family. This enzyme catalyzes the oxidative denitrification of nitroalkane [33]. *Planococcus* sp. PAMC21323 has two nitropropane dioxygenases (Plano_2019 and 2569). It also has three nitroreductases (Plano_226, 336, and 2301) and one azoreductase (AzoR, Plano_0380), which are generally observed in bacteria isolated from soil contaminated with industrial pollutants like trinitrotoluene (dynamite) [34].

For the detoxification of heavy metal ions, *Planococcus* sp. PAMC21323 has three arsenate reductases (Table 5). Plano_1482 and 0840 encoded a low molecular-weight phosphatase (LMWP) family arsenate reductase, whereas Plano_0928 encoded a different type of arsenate reductase from the ArsC family. The LMWP family requires thioredoxin for arsenate reduction, while the ArsC family uses glutaredoxin. It is worth noting that the two LMWP family arsenate reductases are adjacent to the ABC transporter; especially Plano_1482, which was placed together with mercuric ion reductase (Plano_1475) under control of the ArsR family transcription regulator (Plano_1481). In addition, *Planococcus* sp. PAMC21323 also harbors two genes related to tellurite resistance; TelA (Plano_1607) has been established as a determination of tellurite resistance, and the methylase activity of TehB (Plano_1454) has a direct role in tellurite detoxification [35].

### Stress response system of *Planococcus*

Bacteria subjected to bioremediation go through oxidative stress and exhibit high stress resistance because toxic pollutants are metabolized via oxygenase-type enzymes on the catabolic pathway [2]. Accumulation of heavy metal ions strongly induces generation of ROS [36, 37]. In *Pseudomonas*, which has been widely used for bioremediation, transcription of superoxide dismutase (*sod*) genes is induced in the presence of toxic compounds such as phenol, cadmium, and toluene, to remove ROS generated by the compounds [36].

Genome analysis of *Planococcus* sp. PAMC21323 revealed that it has diverse oxidative stress response-related genes (Table 6). To remove superoxide radicals generated from reactions of the various oxygenases, it has two different types of SODs, differentiated by the metal ion cofactor: Mn/Fe SOD (Plano_1316) and Cu/Zn SOD (Plano_2589). Additionally, three catalases, eight peroxiredoxin (Prx) family enzymes, and glutathione peroxidase (GPx) were observed, which are involved in the ROS defense system (Table 6). *Planococcus* sp. PAMC21323 is equipped with one glutaredoxin, eight thioredoxins (TrxA), and three thioredoxin reductases (TrxR) associated with redox balance (Table 6).

**Table 6:** Identified oxidative stress response related genes in PAMC21323

Gene	Product	Locus_tag
Kat	Iron catalase	Plano_0228; Plano_0269
	bifunctional catalase peroxidase	Plano_2972
Sod	Mn/Fe superoxide dismutase	Plano_1316
	Cu/Zn superoxide dismutase	Plano_2589
Prx	Thiol-peroxidase	Plano_1084
	PrxQ (BCP)	Plano_0810
	Prx-like protein	Plano_1452; Plano_1670; Plano_1816; Plano_2134
	Alkyl hydroperoxide reductase C	Plano_2964
	Alkyl hydroperoxide reductase F	Plano_2965
TrxA	Thioredoxin	Plano_0462; Plano_0753; Plano_0826; Plano_0924; Plano_0931; Plano_1054; Plano_1156; Plano_1389; Plano_1669
TrxB	Thioredoxin reductase	Plano_0301; Plano_0900; Plano_1802
Gpx	Glutathione peroxidase	Plano_2887
GR	Glutathione reductase	Plano_2022
Grx	Glutaredoxin	Plano_1634

To reduce thiol-modification in proteins caused by ROS, most microorganisms use low-molecule thiol cofactors. Low-GC gram (+) *Firmicutes* (*Bacillus* and *Staphylococcus*) use the bacillithiol (BSH), and high-GG gram (+) *Actinomycetes* such as *Mycobacterium* produce mycothiol (MSH) [38]. Interestingly, we found that *Planococcus* sp. PAMC21323 has a bifunctional glutathione synthase (Plano_1675), glutathione peroxidase (Plano_2887), and NADPH-dependent glutathione reductase (Plano_2022), involved in glutathione (GSH) maintenance. This indicates that *Planococcus* sp. PAMC21323 has a GSH redox buffer system, and not a bacillithiol-based system, despite its genome similarity with *Bacillus*. Since GSH exhibits a higher capacity to buffer oxidative stress than BSH [39], it may help *Planococcus* sp. PAMC21323 to endure oxidative stress.

Like other psychrophilic bacteria, *Planococcus* sp. PAMC21323 produces a yellow-like pigment as a secondary metabolite. The genome analysis revealed that the pigment is synthesized by a series of genes (Plano_2714~ 2718). In cold environments, pigments can act as modulators of membrane fluidity and maintain proton permeability [40]. Moreover, its antioxidant activity can not only protect the cell against cold and oxidative stress, but also reduce the cytotoxicity of heavy metal ions such as copper [41].

## Conclusion

The genus *Planococcus* grows well under low temperature and high salinity conditions and some *Planococcus* strains are known to have the ability to detoxify pollutants. The psychrotrophic *Planococcus* sp. PAMC21323 was isolated from King George Island in Antarctica. From our analysis of the genome, we identified that *Planococcus* sp. PAMC21323 encodes various genes associated with detoxification of pollutants and possesses a variety of oxidative stress systems to reduce toxicity during bioremediation. Analyzing the genome sequence of *Planococcus* sp. PAMC21323 has shown the potential application of this psychrotrophic strain for bioremediation in harsh environments.

## Additional files

Additional file 1: **Table S1.** List of genes in genomic island regions. (XLSX 14 kb)

## Abbreviations

ANI: Average nucleotide identity
LMWP: Low molecular-weight phosphatase
ROS: Reactive oxygen species
SOD: Superoxide dismutase

## References

[1] Vogt C, Richnow HH (2014). Bioremediation via in situ microbial degradation of organic pollutants. Adv Biochem Eng Biotechnol.

[2] Pandey G, Jain RK (2002). Bacterial chemotaxis toward environmental pollutants: role in bioremediation. Appl Environ Microbiol.

[3] Sengupta D, Sangu K, Shivaji S, Chattopadhyay MK (2015). Tolerance of an Antarctic bacterium to multiple environmental stressors. Curr Microbiol.

[4] Gratia E, Weekers F, Margesin R, D'Amico S, Thonart P, Feller G (2009). Selection of a cold-adapted bacterium for bioremediation of wastewater at low temperatures. Extremophiles.

[5] Michaud L, Lo Giudice A, Saitta M, De Domenico M, Bruni V (2004). The biodegradation efficiency on diesel oil by two psychrotrophic Antarctic marine bacteria during a two-month-long experiment. Mar Pollut Bull.

[6] De Souza MJ, Loka Bharathi PA, Nair S, Chandramohan D (2007). “trade-off” in Antarctic bacteria: limnetic psychrotrophs concede multiple enzyme expressions for multiple metal resistance. Biometals.

[7] Jesus HE, Peixoto RS, Rosado AS (2015). Bioremediation in Antarctic soils. J Pet Environ Biotechnol.

[8] Kocur M, Zdena P, Hodgkiss W, Martinec T (1970). The taxonomic status of the genus *Planococcus* Migula 1894. Int J Syst Bacteriol.

[9] Mykytczuk NC, Wilhelm RC, Whyte LG (2012). *Planococcus halocryophilus* sp. nov., an extreme sub-zero species from high Arctic permafrost. Int J Syst Evol Microbiol.

[10] Lay CY, Mykytczuk NC, Niederberger TD, Martineau C, Greer CW, Whyte LG (2012). Microbial diversity and activity in hypersaline high Arctic spring channels. Extremophiles.

[11] Mykytczuk NC, Foote SJ, Omelon CR, Southam G, Greer CW, Whyte LG (2013). Bacterial growth at −15 degrees C; molecular insights from the permafrost bacterium *Planococcus halocryophilus* Or1. ISME J.

[12] Hupert-Kocurek K, Guzik U, Wojcieszynska D (2012). Characterization of catechol 2,3-dioxygenase from *Planococcus* sp. strain S5 induced by high phenol concentration. Acta Biochim Pol.

[13] Li H, Liu YH, Luo N, Zhang XY, Luan TG, Hu JM, Wang ZY, Wu PC, Chen MJ, Lu JQ (2006). Biodegradation of benzene and its derivatives by a psychrotolerant and moderately haloalkaliphilic *Planococcus* sp. strain ZD22. Res Microbiol.

[14] Tamura K, Peterson D, Peterson N, Stecher G, Nei M, Kumar S (2011). MEGA5: molecular evolutionary genetics analysis using maximum likelihood, evolutionary distance, and maximum parsimony methods. Mol Biol Evol.

[15] Reddy GS, Prakash JS, Vairamani M, Prabhakar S, Matsumoto GI, Shivaji S (2002). *Planococcus antarcticus* and *Planococcus psychrophilus* spp. nov. isolated from cyanobacterial mat samples collected from ponds in Antarctica. Extremophiles.

[16] Pagani I, Liolios K, Jansson J, Chen IM, Smirnova T, Nosrat B, Markowitz VM, Kyrpides NC (2012). The genomes OnLine database (GOLD) v.4: status of genomic and metagenomic projects and their associated metadata. Nucleic Acids Res.

[17] Aziz RK, Bartels D, Best AA, DeJongh M, Disz T, Edwards RA, Formsma K, Gerdes S, Glass EM, Kubal M (2008). The RAST server: rapid annotations using subsystems technology. BMC Genomics.

[18] Zdobnov EM, Apweiler R (2001). InterProScan--an integration platform for the signature-recognition methods in InterPro. Bioinformatics.

[19] Mistry J, Finn R (2007). Pfam: a domain-centric method for analyzing proteins and proteomes. Methods Mol Biol.

[20] Lowe TM, Eddy SR (1997). tRNAscan-SE: a program for improved detection of transfer RNA genes in genomic sequence. Nucleic Acids Res.

[21] Finn RD, Clements J, Eddy SR (2011). HMMER web server: interactive sequence similarity searching. Nucleic Acids Res.

[22] Zhou Y, Liang Y, Lynch KH, Dennis JJ, Wishart DS (2011). PHAST: a fast phage search tool. Nucleic Acids Res.

[23] Hsiao W, Wan I, Jones SJ, Brinkman FS (2003). IslandPath: aiding detection of genomic islands in prokaryotes. Bioinformatics.

[24] Krogh A, Larsson B, von Heijne G, Sonnhammer EL (2001). Predicting transmembrane protein topology with a hidden Markov model: application to complete genomes. J Mol Biol.

[25] Petersen TN, Brunak S, von Heijne G, Nielsen H (2011). SignalP 4.0: discriminating signal peptides from transmembrane regions. Nat Methods.

[26] Goris J, Konstantinidis KT, Klappenbach JA, Coenye T, Vandamme P, Tiedje JM (2007). DNA-DNA hybridization values and their relationship to whole-genome sequence similarities. Int J Syst Evol Microbiol.

[27] Juhas M, van der Meer JR, Gaillard M, Harding RM, Hood DW, Crook DW (2009). Genomic islands: tools of bacterial horizontal gene transfer and evolution. FEMS Microbiol Rev.

[28] Janssen BD, Garza-Sanchez F, Hayes CS (2015). YoeB toxin is activated during thermal stress. Microbiology.

[29] Arnaouteli S, Giastas P, Andreou A, Tzanodaskalaki M, Aldridge C, Tzartos SJ, Vollmer W, Eliopoulos E, Bouriotis V (2015). Two putative polysaccharide deacetylases are required for osmotic stability and cell shape maintenance in *Bacillus anthracis*. J Biol Chem.

[30] Lipscomb JD (2008). Mechanism of extradiol aromatic ring-cleaving dioxygenases. Curr Opin Struct Biol.

[31] Hayes RP, Green AR, Nissen MS, Lewis KM, Xun L, Kang C (2013). Structural characterization of 2,6-dichloro-p-hydroquinone 1,2-dioxygenase (PcpA) from *Sphingobium chlorophenolicum*, a new type of aromatic ring-cleavage enzyme. Mol Microbiol.

[32] Conaway CC, Nie G, Hussain NS, Fiala ES (1991). Comparison of oxidative damage to rat liver DNA and RNA by primary nitroalkanes, secondary nitroalkanes, cyclopentanone oxime, and related compounds. Cancer Res.

[33] Ha JY, Min JY, Lee SK, Kim HS, Kim DJ, Kim KH, Lee HH, Kim HK, Yoon HJ, Suh SW (2006). Crystal structure of 2-nitropropane dioxygenase complexed with FMN and substrate. Identification of the catalytic base. J Biol Chem.

[34] Caballero A, Lazaro JJ, Ramos JL, Esteve-Nunez A (2005). PnrA, a new nitroreductase-family enzyme in the TNT-degrading strain *Pseudomonas putida* JLR11. Environ Microbiol.

[35] Anantharaman V, Iyer LM, Aravind L (2012). Ter-dependent stress response systems: novel pathways related to metal sensing, production of a nucleoside-like metabolite, and DNA-processing. Mol BioSyst.

[36] Kim J, Park W (2014). Oxidative stress response in Pseudomonas putida. Appl Microbiol Biotechnol.

[37] Park W, Jeon CO, Cadillo H, DeRito C, Madsen EL (2004). Survival of naphthalene-degrading *Pseudomonas putida* NCIB 9816-4 in naphthalene-amended soils: toxicity of naphthalene and its metabolites. Appl Microbiol Biotechnol.

[38] Loi VV, Rossius M, Antelmann H (2015). Redox regulation by reversible protein S-thiolation in bacteria. Front Microbiol.

[39] Sharma SV, Arbach M, Roberts AA, Macdonald CJ, Groom M, Hamilton CJ (2013). Biophysical features of bacillithiol, the glutathione surrogate of Bacillus subtilis and other firmicutes. Chembiochem.

[40] Chintalapati S, Kiran MD, Shivaji S (2004). Role of membrane lipid fatty acids in cold adaptation. Cell Mol Biol.

[41] Irazusta V, Nieto-Penalver CG, Cabral ME, Amoroso MJ, de Figueroa LIC (2013). Relationship among carotenoid production, copper bioremediation and oxidative stress in *Rhodotorula mucilaginosa* RCL-11. Process Biochem.

[42] Field D, Garrity G, Gray T, Morrison N, Selengut J, Sterk P, Tatusova T, Thomson N, Allen MJ, Angiuoli SV (2008). The minimum information about a genome sequence (MIGS) specification. Nat Biotechnol.

[43] Woese CR, Kandler O, Wheelis ML (1990). Towards a natural system of organisms: proposal for the domains archaea, Bacteria, and Eucarya. Proc Natl Acad Sci U S A.

[44] Gibbons NE, Murray RGE (1978). Proposals concerning the higher taxa of Bacteria. Int J Syst Evol Microbiol.

[45] Euzeby J (2010). Validation list no. 132. List of new names and new combinations previously effectively, but not validly, published. Int J Syst Evol Microbiol.

[46] Skerman VBD, McGowan V, Sneath PHA (1980). Approved lists of bacterial names. Int J Syst Evol Microbiol.

[47] Ashburner M, Ball CA, Blake JA, Botstein D, Butler H, Cherry JM, Davis AP, Dolinski K, Dwight SS, Eppig JT (2000). Gene ontology: tool for the unification of biology. The Gene Ontology Consortium. Nat Genet.

